# Supplementary material for: Mechanisms governing the pioneering and redistribution capabilities of the non-classical pioneer PU.1
Source: Nat Commun. 2020 Jan 21;11:402. doi: 10.1038/s41467-019-13960-2 (PMC6972792; doi:10.1038/s41467-019-13960-2)
Supplement: Supplementary file 7 — Source data [file 41467_2019_13960_MOESM7_ESM.zip › Source_Data/Figure6/Figure6B_MotifScanOutput/ETS1_PU.1/homerResults/motif3.similar.html]

motif3

## Information for motif3

A
G
T
C
C
G
T
A
A
G
T
C
A
C
G
T
A
C
G
T
A
G
T
C
A
G
C
T
  
Reverse Opposite:  

C
T
G
A
A
C
T
G
C
G
T
A
C
G
T
A
A
C
T
G
A
C
G
T
C
T
A
G
  

|  |  |
| --- | --- |
| p-value: | 1e-865 |
| log p-value: | -1.994e+03 |
| Information Content per bp: | 1.929 |
| Number of Target Sequences with motif | 6009.0 |
| Percentage of Target Sequences with motif | 37.96% |
| Number of Background Sequences with motif | 3980.4 |
| Percentage of Background Sequences with motif | 12.41% |
| Average Position of motif in Targets | 101.5 +/- 55.1bp |
| Average Position of motif in Background | 101.4 +/- 69.9bp |
| Strand Bias (log2 ratio + to - strand density) | 0.0 |
| Multiplicity (# of sites on avg that occur together) | 1.07 |
| Motif File: | file (matrix) reverse opposite |

### Similar de novo motifs found

|  |  |  |  |  |  |  |  |
| --- | --- | --- | --- | --- | --- | --- | --- |
| Rank | Match Score | Redundant Motif | P-value | log P-value | % of Targets | % of Background | Motif file |
| 1 | 0.802 | C T G A A T G C C G A T A C G T A G T C C G A T A T C G G A T C G C A T G C A T G C A T | 1e-137 | -317.129684 | 8.19% | 2.89% | motif file (matrix) |
| 2 | 0.833 | C G T A A T G C C G A T A C G T A G T C A C G T A G T C | 1e-36 | -84.950385 | 13.04% | 9.18% | motif file (matrix) |
| 3 | 0.936 | G A T C C G T A A G T C A C G T A C G T A G T C A C G T G T A C | 1e-35 | -80.864120 | 6.18% | 3.62% | motif file (matrix) |
